# Supplementary material for: A cosmic-ray loaded nascent outflow driven by a massive star cluster
Source: Nat Commun. 2025 Dec 9;16:10820. doi: 10.1038/s41467-025-65592-4 (PMC12690131; doi:10.1038/s41467-025-65592-4)
Supplement: Supplementary file 1 — Supplementary Information [file 41467_2025_65592_MOESM1_ESM.pdf]

A cosmic-ray loaded nascent outflow driven by a massive star cluster

Supplementary Material

Marianne Lemoine-Goumard[1], Lucia Härer[2], Lars Mohrmann[2], Romain Bernet[3,4], Jim Hinton[2], Giada Peron[5], Brian Reville[2], Luigi Tibaldo[3], Thibault Vieu[2]

[1]Université Bordeaux, CNRS, LP2I Bordeaux, UMR 5797, F-33170 Gradignan, France.
[2]Max-Planck-Institut für Kernphysik, Saupfercheckweg 1, D-69117 Heidelberg, Germany.
[3]IRAP, Université de Toulouse, CNRS, CNES, UPS, F-31028 Toulouse, France.
[4]Institut Supérieur de l'Aéronautique et de l'Espace (ISAE-SUPAERO), Université de Toulouse, F-31055 Toulouse, France.
[5]INAF Osservatorio Astrofisico Arcetri, Largo E. Fermi, 5, 50125,Florence, Italy.

Corresponding authors: lemoine@cenbg.in2p3.fr; lucia.haerer@mpi-hd.mpg.de; lars.mohrmann@mpi-hd.mpg.de;

Further details on the *Fermi*-LAT data analysis

Handling of energy dispersion

We account for the effect of energy dispersion (the reconstructed energy being different from the true energy due to finite detector resolution) by setting the parameter `edisp_bins` $= -1$. With this setting, the energy dispersion correction operates on the spectra with one extra bin below and above the threshold of the analysis. The energy dispersion correction is applied to all sources in the model, except for the isotropic diffuse emission model. More details can be found in the FSSC: https://fermi.gsfc.nasa.gov/ssc/data/analysis/documentation/Pass8_edisp_usage.html.

Background modelling

For more details on the galactocentric templates of the Galactic diffuse emission model, see https://fermi.gsfc.nasa.gov/ssc/data/analysis/software/aux/4fgl/Galactic_Diffuse_Emission_Model_for_the_4FGL_Catalog_Analysis.pdf. The free parameters chosen to obtain a good fit to the data after inspection of the residual maps are the following: for the CO and H I components, we leave free both normalisation and shape for the closest rings (4, 5, 6), only the normalisation for some others (2, 3, 7, 8, 9), and completely removed rings 0 and 1 which do not contribute for this line of sight. We merged all rings for the IC component and leave free its normalisation and shape. The isotropic diffuse emission model is available at https://fermi.gsfc.nasa.gov/ssc/data/access/lat/BackgroundModels.html.

Initial re-optimisation

Starting from the 4FGL-DR4 baseline model, our analysis starts with a preliminary optimisation using the `optimize` function provided by *fermipy*. In this optimisation step, we first fit the spectral parameters of the different Galactic interstellar emission components and the isotropic background together with the normalisation of the five brightest sources. Then, we individually fit the normalisations of all sources inside the region of interest (ROI) in the order of their total predicted counts in the model ($N_{\rm pred}$) down to $N_{\rm pred} = 10$. The optimisation is concluded by individually fitting the index and normalisation parameters of all sources with a test statistic (TS) value above 16 starting from the highest TS sources. This TS value is determined from the first two steps of the ROI optimisation by $\mathrm{TS} = 2(\ln \mathcal{L}_1 - \ln \mathcal{L}_0)$, where $\mathcal{L}_0$ and $\mathcal{L}_1$ are the likelihoods of the null hypothesis (background only) and the hypothesis being tested (source plus background). This optimisation is followed by a second one where the number of bright sources fit together with the diffuse backgrounds is increased to 10. We then check if additional sources need to be added in the ROI. For this purpose, we compute a TS map that tests at each pixel the significance of a source with a generic E^{-2} spectrum against the background-only hypothesis. We iteratively add 6 point sources in the model where the TS exceeded 25. They are all located more than two degrees away from Westerlund 1; their positions and TS are reported in Supplementary Table 1.

Supplementary Table 1: **New *Fermi*-LAT point-like sources.** Coordinates and TS values of the six point sources added in the region of interest.

Source name	Galactic coordinates (l, b)	TS
PS J1617.2−5106	(332.36°, −0.39°)	58
PS J1633.7−4754	(336.50°, −0.06°)	48
PS J1636.2−4659	(337.46°, 0.25°)	32
PS J1708−4104	(345.64°, −0.38°)	31
PS J1708.9-3941	(346.88°, 0.28°)	30
PS J1710−3921	(347.27°, 0.32°)	39

Spectral modelling

For each component of our best spatial model, that is, for both halves of the H.E.S.S. TeV template and for both halves of the Gaussian model for J1654−467, we fit a power-law (PL) model as well as a power-law model with exponential cut-off (PLcut). The improvement between the PL model and the PLcut model is tested using a likelihood ratio test (with test statistic $\mathrm{TS_{cut}} = 2(\ln \mathcal{L}_{\mathrm{PLcut}} - \ln \mathcal{L}_{\mathrm{PL}})$). Supplementary Table 2 summarises the spectral modelling results.

We then derive spectral points (spectral energy distribution, or SED) for each component in the 3 GeV – 3 TeV energy range in 6 logarithmically-spaced energy bins by performing a maximum likelihood spectral analysis to estimate the photon flux in each interval, assuming a power-law shape with fixed photon index Γ=2 for the source of interest. The normalisations of the closest H I and CO galactocentric rings and the isotropic emission are left free in each energy bin as well as those of the sources within 2°. A 95% confidence level upper limit is computed when the TS value is lower than 1. A comparison of the energy spectra obtained for the near and far part of J1654−467 is displayed in Supplementary Fig. 1.

Supplementary Table 2: **Spectral parameters of split *Fermi*-LAT source templates.** The first two rows detail the parameters for the two halves of the H.E.S.S. TeV emission template for the region close to Westerlund 1; the last two rows refer to the spectral models fitted in the near and far region of J1654−467, respectively. The first and second error represent statistical and systematic error, respectively. Columns 4 and 5 provide the component's TS value and the improvement of the exponential cut-off representation with respect to the PL model $\mathrm{TS_{cut}}$.

Source name	Spectral index	Energy flux (3 GeV – 3 TeV) (10^{-11} erg cm^{-2} s^{-1})	Cut-off (GeV)	TS	$\mathrm{TS_{cut}}$
Wd1 (region 1)	$2.2 \pm 0.02 \pm 0.1$	$4.9 \pm 0.4 \pm 0.2$		256	0
Wd1 (region 2)	$2.0 \pm 0.1 \pm 0.3$	$1.8 \pm 0.5 \pm 0.2$		31	2
J1654−467 (near)	$2.0 \pm 0.02 \pm 0.2$	$12.4 \pm 0.8 \pm 0.2$		551	2
J1654−467 (far)	$1.8 \pm 0.1 \pm 0.3$	$6.6 \pm 0.5 \pm 0.6$	$149 \pm 67 \pm 61$	487	12

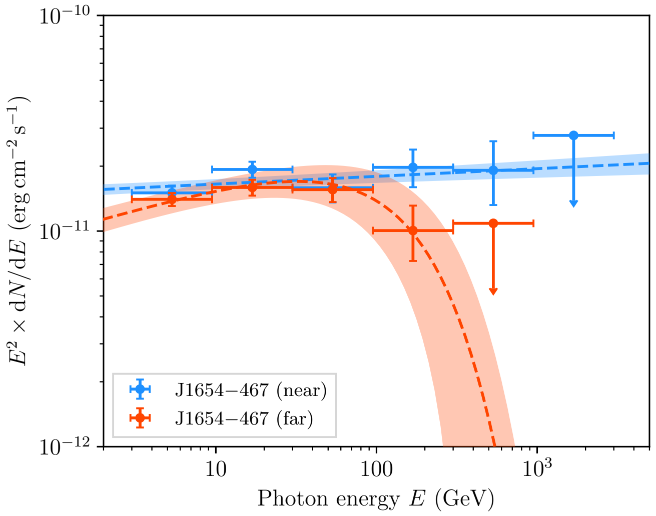

Supplementary Fig. 1: *Fermi*-LAT SEDs of the near and far part of J1654−467. The dashed lines show the preferred spectral model, which is a power law for the near part and a power law with exponential cut-off for the far part (cf. Supplementary Table 2). The shaded bands display the statistical uncertainty. Error bars on the flux points denote 68% c.l. statistical uncertainties; upper limits are at 95% c.l.

Systematic uncertainty estimation

We show in Supplementary Fig. 2 the SEDs of the four main model components including their associated systematic uncertainties. The systematic errors include uncertainties on the Galactic diffuse emission model and on the instrument's effective area. The former are calculated using eight alternative diffuse emission models following the same procedure as in the first *Fermi*-LAT supernova remnant catalogue [1], while the latter are obtained by applying two scaling functions on the effective area following the standard method defined in [2]. These two sources of systematic uncertainties are added in quadrature to represent the total systematic uncertainty.

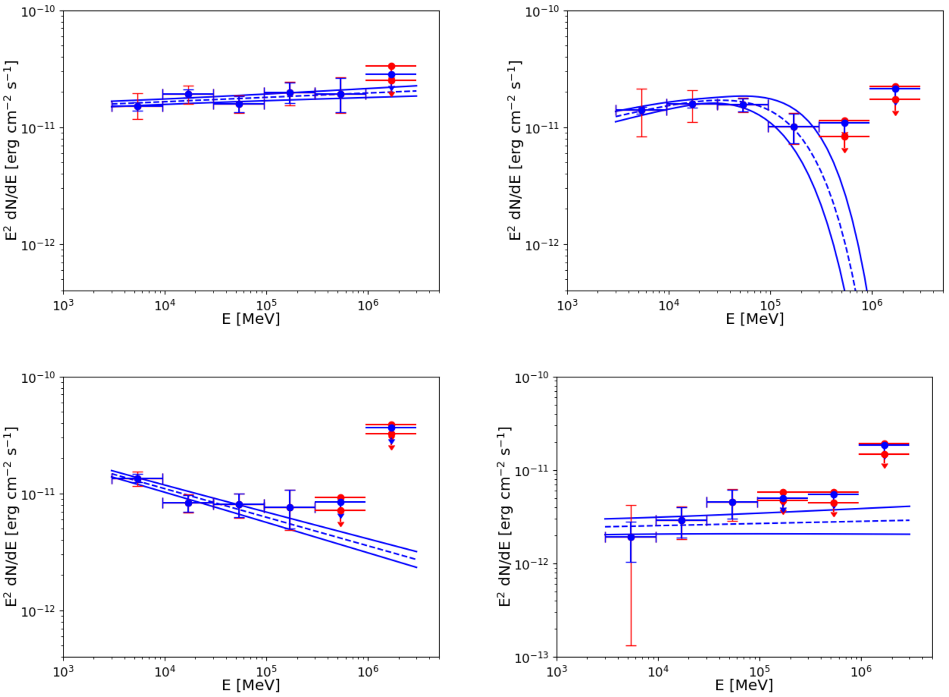

Supplementary Fig. 2: *Fermi*-LAT SEDs including systematic uncertainties. Top row: SEDs for the near (left) and far (right) part of J1654−467. Bottom row: SEDs for the emission surrounding Westerlund 1, in region 1 (left) and region 2 (right). For all SEDs, blue error bars represent 68% c.l. statistical uncertainties, while the red ones correspond to the statistical and systematic uncertainties added in quadrature. For upper limits, the two red arrows indicate the extrema of upper limits obtained with the different systematics. The solid and dashed blue lines represent the best spectral fit and its 68% confidence band. All spectral parameters are reported in Supplementary Table 2.

Molecular gas column densities in the J1654–467 region

We also searched for the presence of dense molecular hydrogen gas at the position of J1654−467. To this purpose, we use observations of the ^{12}CO (1–0) emission line [3] – a commonly employed tracer for molecular hydrogen – and generate brightness temperature maps for our region of interest. The maps in Supplementary Fig. 3, shown for the same velocity ranges as used in Fig. 3 of the main article, clearly show that no dense molecular gas in spatial correlation with the GeV emission measured with *Fermi*-LAT is present.

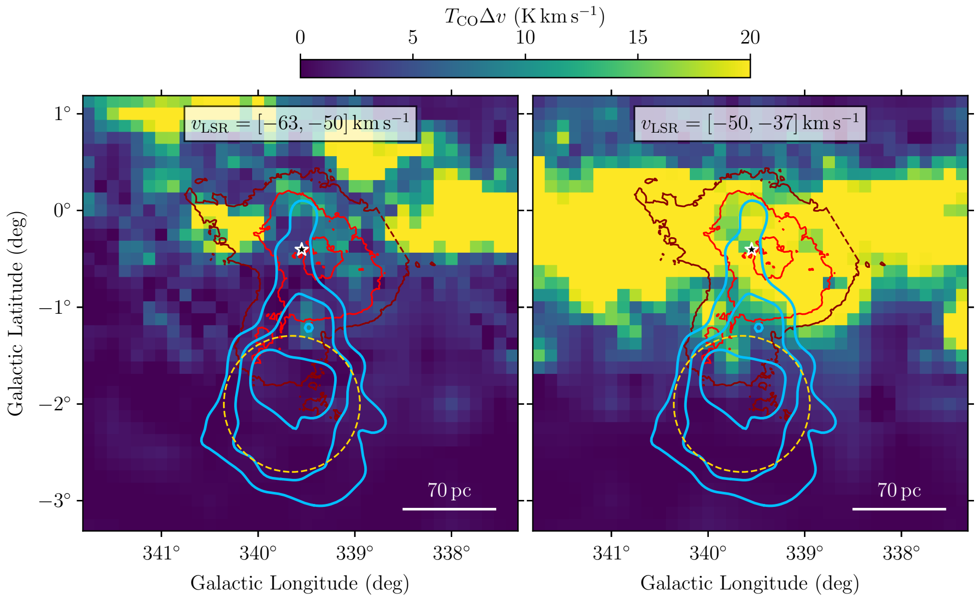

Supplementary Fig. 3: Brightness temperature of the ^{12}CO (1–0) emission line in the region of interest [3]. Ranges in velocity with respect to the local standard of rest are indicated in each panel. CO is a tracer for molecular hydrogen gas. *Fermi*-LAT GeV emission contours (blue), extent of J1654−467 (dashed orange), H.E.S.S. TeV flux contours (red), and position of Westerlund 1 (star marker) are the same as in Fig. 3 of the main article.

Radio synchrotron limits

The population of electrons that is responsible for the gamma-ray emission of J1654−467 is expected to also emit synchrotron radiation in the presence of a magnetic field. While this synchrotron radiation is negligible in the gamma-ray regime for the magnetic field strengths adopted in our model (cf. Fig. 9 in the main article), it could in principle be detectable at smaller wavelengths. As this possibility has already been considered for the immediate surroundings of Westerlund 1 [4, 5], we focus here on the region of J1654−467. We investigate publicly available radio continuum maps at five different frequencies (45 MHz [6, 7], 150 MHz [7, 8], 408 MHz [9, 10], 1.42 GHz [11, 12], 22.8 GHz [13]) but did not find any features related to the outflow region. We therefore derive upper limits by integrating the intensity within the yellow dashed circle shown in Fig. 1 of the main article; these limits are shown together with the GeV and TeV flux points and our model curves in Supplementary Fig. 4.

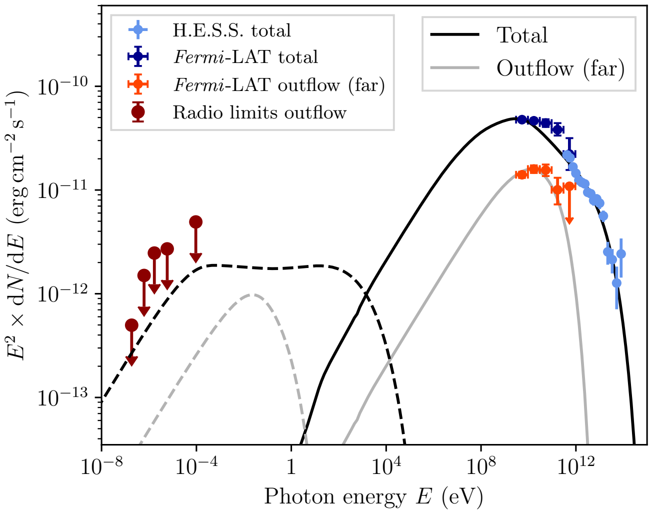

Supplementary Fig. 4: Spectral energy distribution and model curves as shown in Fig. 4 of the main article, but including synchrotron model components and limits for the outflow region in the radio domain. See text for details about the radio limits.

References

[1] Acero, F., *et al.* (*Fermi*-LAT Collaboration): The First Fermi LAT Supernova Remnant Catalog. Astrophysical Journal Supplement Series **224**, 8 (2016) DOI: 10.3847/0067-0049/224/1/8 arXiv:1511.06778

[2] Ackermann, M., *et al.* (*Fermi*-LAT Collaboration): The Fermi Large Area Telescope on Orbit: Event Classification, Instrument Response Functions, and Calibration. Astrophysical Journal Supplement Series **203**, 4 (2012) DOI: 10.1088/0067-0049/203/1/4 arXiv:1206.1896

[3] Dame, T.M., Hartmann, D., Thaddeus, P.: The Milky Way in Molecular Clouds: A New Complete CO Survey. Astrophysical Journal **547**, 792 (2001) DOI: 10.1086/318388 arXiv:astro-ph/0009217

[4] Aharonian, F., *et al.* (H.E.S.S. Collaboration): A deep spectromorphological study of the γ-ray emission surrounding the young massive stellar cluster Westerlund 1. Astronomy & Astrophysics **666**, 124 (2022) DOI: 10.1051/0004-6361/202244323 arXiv:2207.10921

[5] Härer, L.K., Reville, B., Hinton, J., Mohrmann, L., Vieu, T.: Understanding the TeV γ-ray emission surrounding the young massive star cluster Westerlund 1. Astronomy & Astrophysics **671**, 4 (2023) DOI: 10.1051/0004-6361/202245444 arXiv:2301.10496

[6] Guzmán, A.E., May, J., Alvarez, H., Maeda, K.: All-sky Galactic radiation at 45 MHz and spectral index between 45 and 408 MHz. Astronomy & Astrophysics **525**, 138 (2011) DOI: 10.1051/0004-6361/200913628 arXiv:1011.4298

[7] Monsalve, R.A., *et al.*: Absolute Calibration of Diffuse Radio Surveys at 45 and 150 MHz. Astrophysical Journal **908**, 145 (2021) DOI: 10.3847/1538-4357/abd558 arXiv:2012.11019

[8] Landecker, T.L., Wielebinski, R.: The Galactic Metre Wave Radiation: A two-frequency survey between declinations $+25°$ and $-25°$ and the preparation of a map of the whole sky. Australian Journal of Physics, Astrophysical Supplement **16**, 1 (1970)

[9] Remazeilles, M., Dickinson, C., Banday, A.J., Bigot-Sazy, M.-A., Ghosh, T.: An improved source-subtracted and destriped 408-MHz all-sky map. Monthly Notices of the Royal Astronomical Society **451**, 4311–4327 (2015) DOI: 10.1093/mnras/stv1274 arXiv:1411.3628

[10] Orlando, E.: Imprints of cosmic rays in multifrequency observations of the interstellar emission. Monthly Notices of the Royal Astronomical Society **475**, 2724–2742 (2018) DOI: 10.1093/mnras/stx3280 arXiv:1712.07127

[11] Testori, J.C., *et al.*: A radio continuum survey of the southern sky at 1420 MHz: Observations and data reduction. Astronomy & Astrophysics **368**, 1123–1132 (2001) DOI: 10.1051/0004-6361:20010088 arXiv:astro-ph/0101322

[12] Reich, P., Testori, J.C., Reich, W.: A radio continuum survey of the southern sky at 1420 MHz: The atlas of contour maps. Astronomy & Astrophysics **376**, 861–877 (2001) DOI: 10.1051/0004-6361:20011000

[13] Planck Collaboration: Planck 2015 results. X. Diffuse component separation: Foreground maps. Astronomy & Astrophysics **594**, 10 (2016) DOI: 10.1051/0004-6361/201525967 arXiv:1502.01588
